# Supplementary figures and images for: Distinguishing between recruitment and spread of silent chromatin structures in Saccharomyces cerevisiae
Source: eLife. 2022 Jan 24;11:e75653. doi: 10.7554/eLife.75653 (PMC8830885; doi:10.7554/eLife.75653)

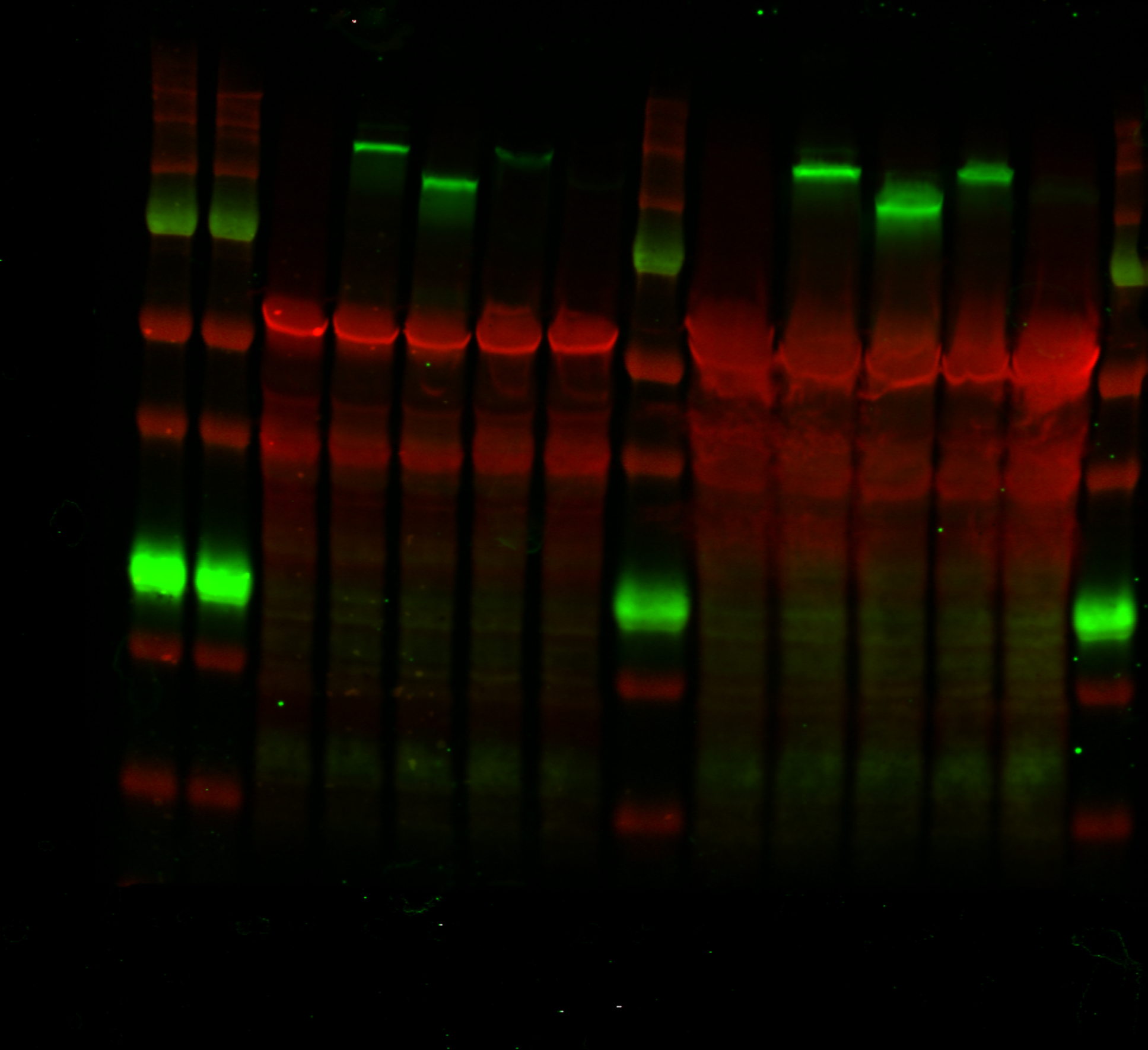

Supplement: Figure 2—source data 1. [file elife-75653-fig2-data1.zip › Figure 2-source data 1_unedited.tif]

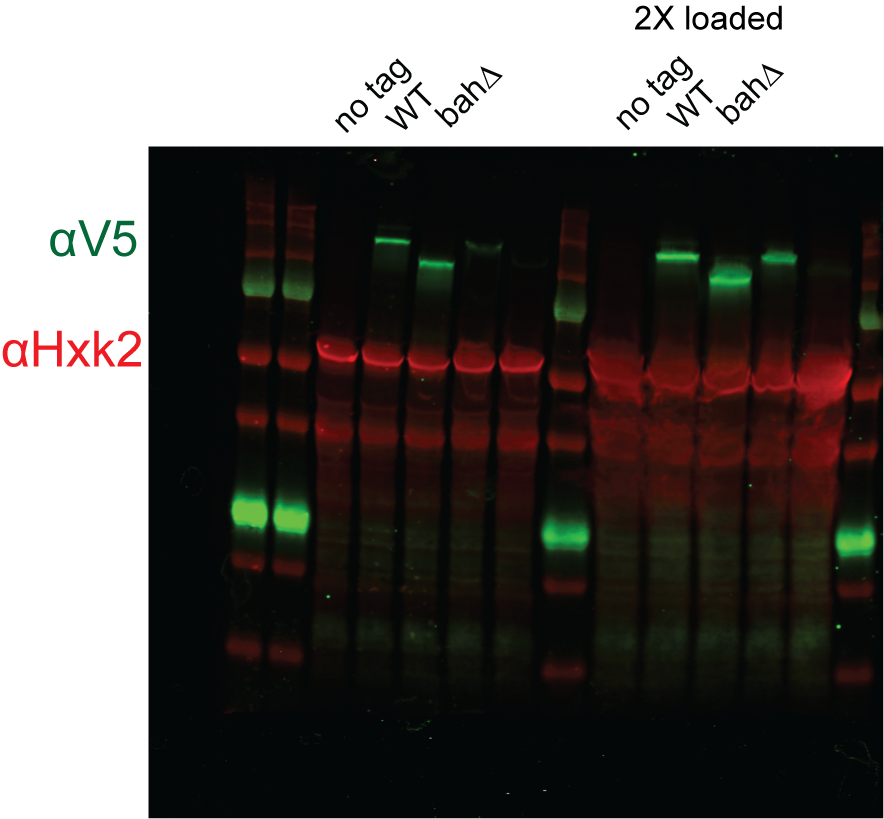

Supplement: Figure 2—source data 1. [file elife-75653-fig2-data1.zip › Figure 2-source data 1_labelled.tif]

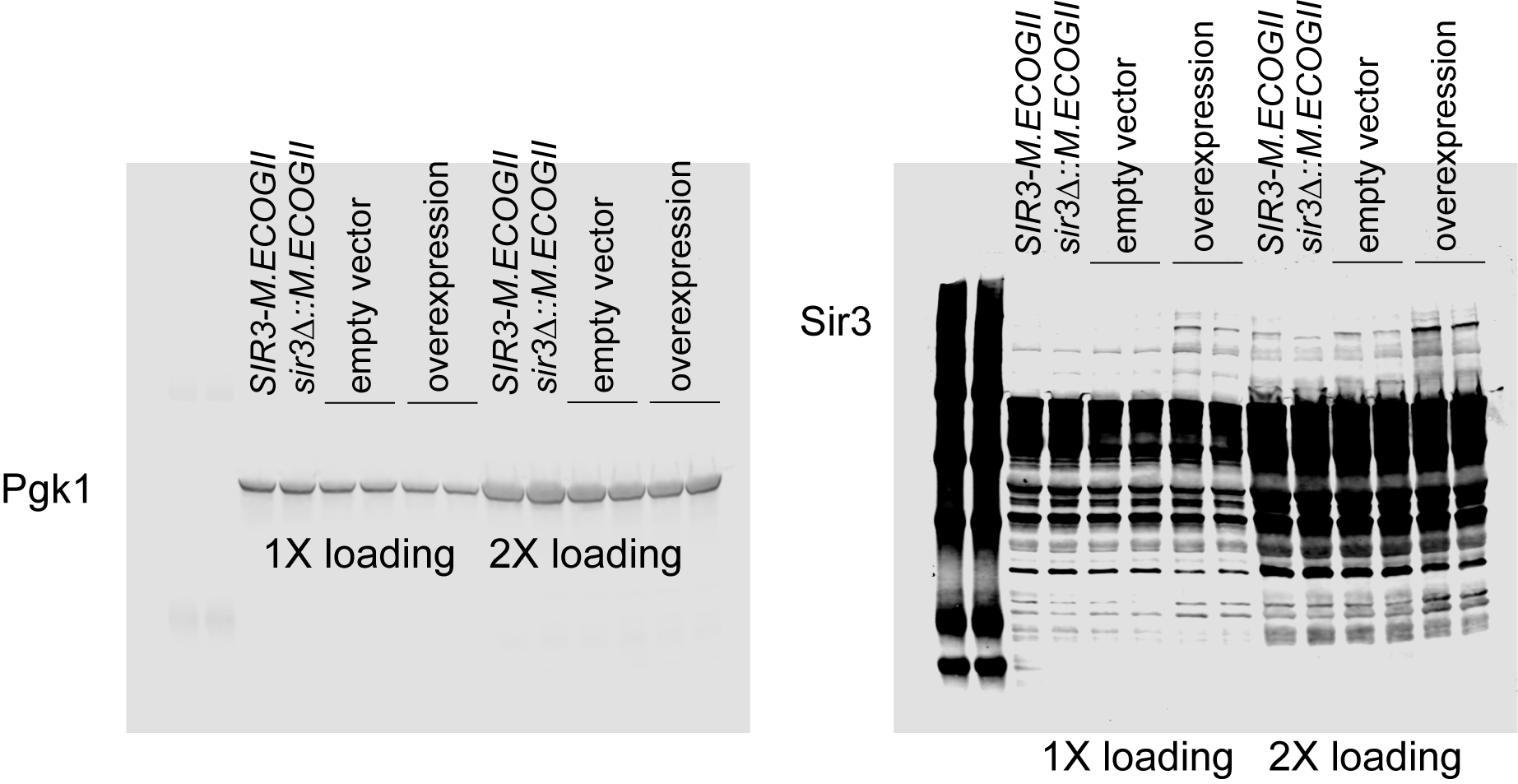

Supplement: Figure 3—source data 1. [file elife-75653-fig3-data1.zip › 220114_labelled.tif]

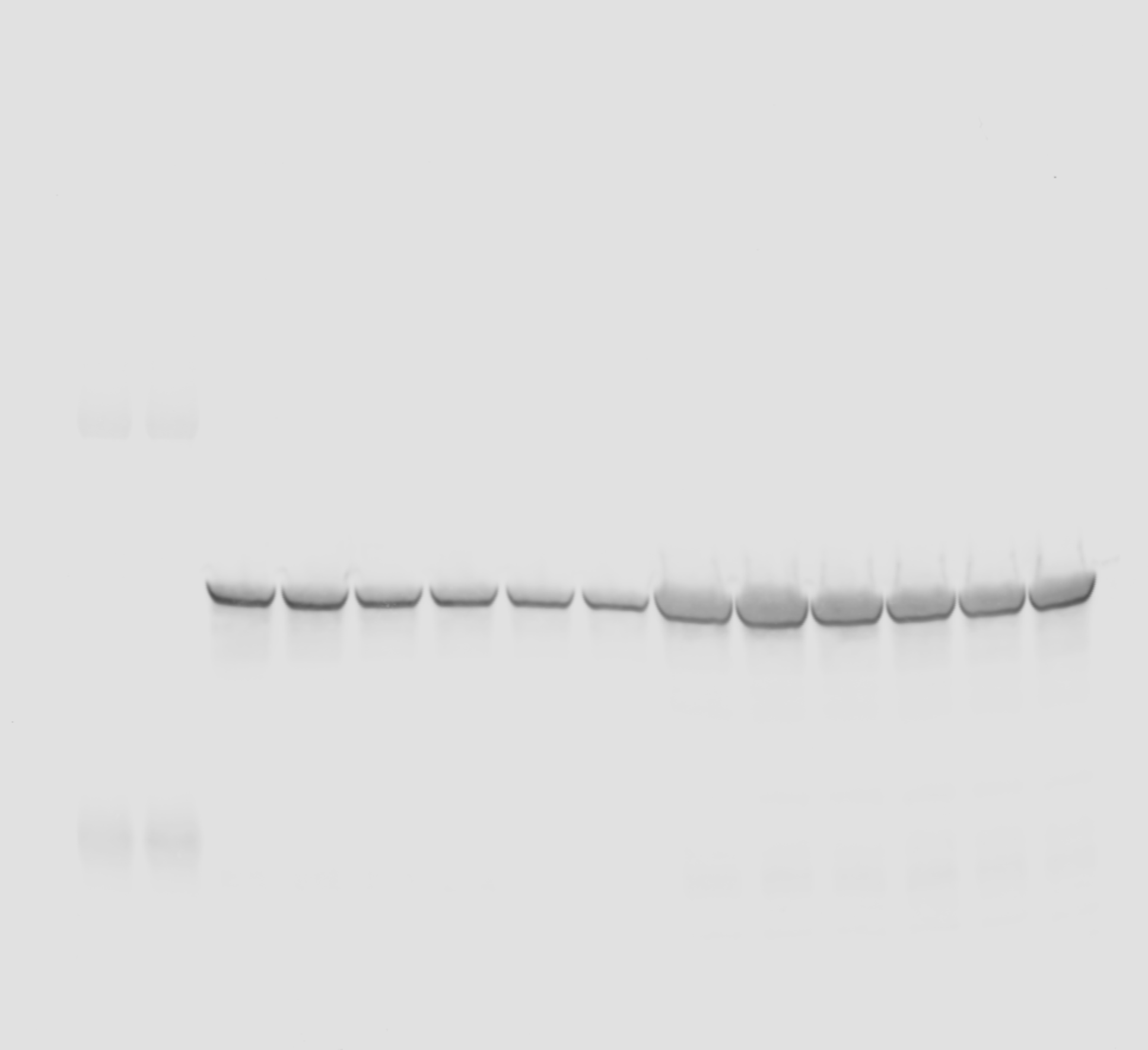

Supplement: Figure 3—source data 1. [file elife-75653-fig3-data1.zip › 220114_Pgk1-bnw-unedited.tif]

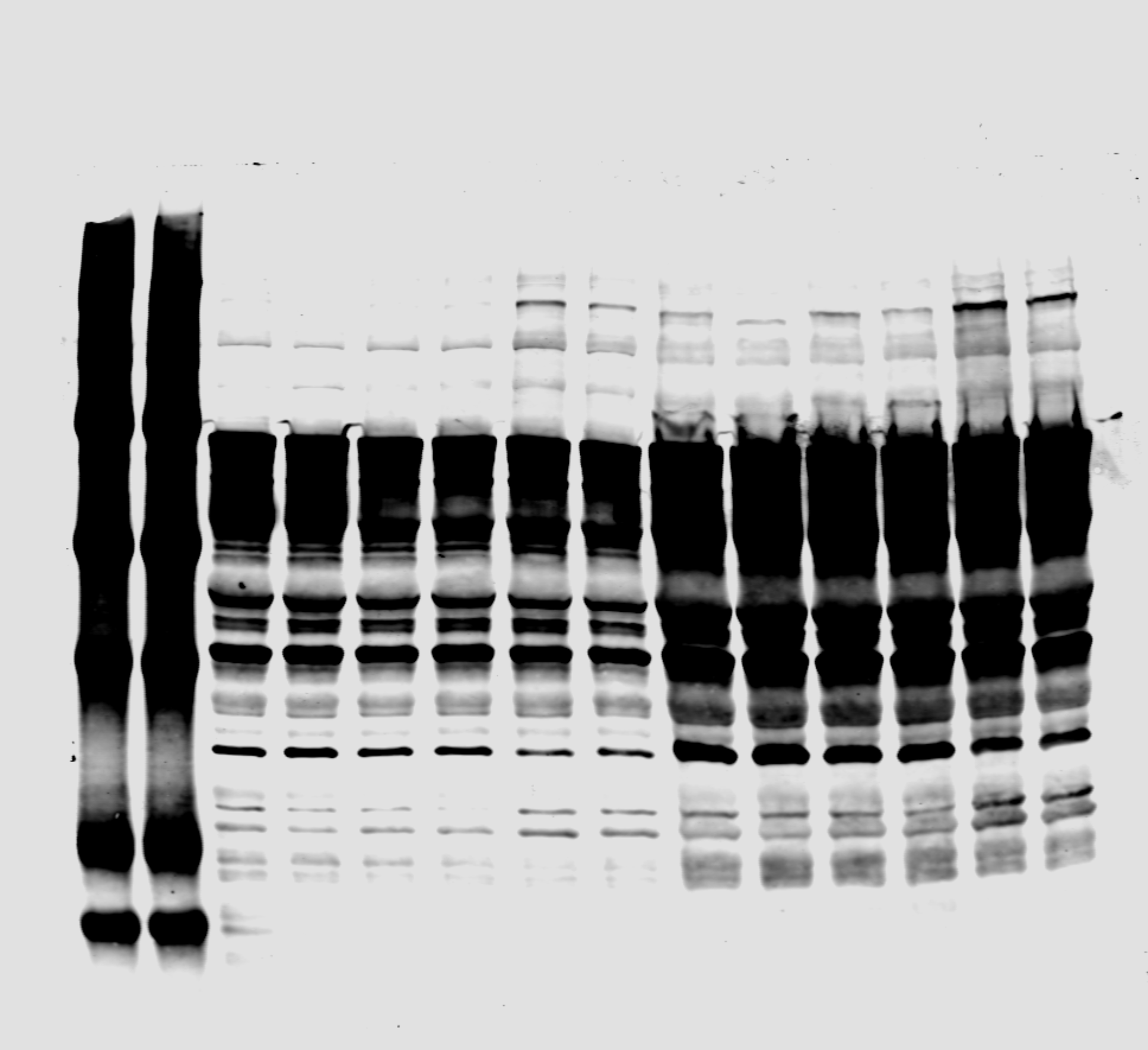

Supplement: Figure 3—source data 1. [file elife-75653-fig3-data1.zip › 220114_Sir3-bnw-unedited.tif]
