# Supplementary material for: Distinguishing between recruitment and spread of silent chromatin structures in Saccharomyces cerevisiae
Source: eLife. 2022 Jan 24;11:e75653. doi: 10.7554/eLife.75653 (PMC8830885; doi:10.7554/eLife.75653)
Supplement: Source code 5. [file elife-75653-code5.zip › Fig1.html]

Fig1


# Fig1

#### Molly Brothers

#### 7/19/2021

# Main Figure

## SIR2/3/4-M.ECOGII aggregate methylation with nanopore sequencing

```
# LOESS PLOT FUNCTION
# d = data, ch = chromosome, b = beginning, e = end
plot_loess <- function(d0, d1, d2, d3, ch, b, e){
  region_methyl_0 <- d0[chrom == ch & start > b & start < e]
  lo_methyl_0 <- loess(percentage ~ start, data = region_methyl_0, weights = region_methyl_0$coverage, enp.target = 100)
  
  region_methyl_1 <- d1[chrom == ch & start > b & start < e]
  lo_methyl_1 <- loess(percentage ~ start, data = region_methyl_1, weights = region_methyl_1$coverage, enp.target = 100)
  
  region_methyl_2 <- d2[chrom == ch & start > b & start < e]
  lo_methyl_2 <- loess(percentage ~ start, data = region_methyl_2, weights = region_methyl_2$coverage, enp.target = 100)
  
  region_methyl_3 <- d3[chrom == ch & start > b & start < e]
  lo_methyl_3 <- loess(percentage ~ start, data = region_methyl_3, weights = region_methyl_3$coverage, enp.target = 100)
  
  plot(x=lo_methyl_0$x[order(lo_methyl_0$x)], y=lo_methyl_0$fitted[order(lo_methyl_0$x)],
       type = 'l', lwd=2, ylim = c(0,100), col = "black", frame.plot = FALSE,
       xlab = "", ylab = "")
  lines(x=lo_methyl_1$x[order(lo_methyl_1$x)], y=lo_methyl_1$fitted[order(lo_methyl_1$x)],
        type = 'l', lwd=2, ylim = c(0,100), col = "mediumpurple4")
  lines(x=lo_methyl_2$x[order(lo_methyl_2$x)], y=lo_methyl_2$fitted[order(lo_methyl_2$x)],
        type = 'l', lwd=2, ylim = c(0,100), col = "forestgreen")
  lines(x=lo_methyl_3$x[order(lo_methyl_3$x)], y=lo_methyl_3$fitted[order(lo_methyl_3$x)],
        type = 'l', lwd=2, ylim = c(0,100), col = "deeppink")
  box(bty="l")
}

# DATA
columns <- c("chrom", "start", "end", "name", "score", 
             "strand", "startCodon", "stopCodon", "color", 
             "coverage", "percentage")
select_cols <- c("chrom", "start", "coverage", "percentage")

# sir3∆::EcoGII
methyl_0 <- fread("/home/mbrothers/nanopore/201125_Turkey/modified_bases.aggregate05.6mA.bed",
                col.names = columns)
methyl_filtered_0 <- methyl_0[coverage > 10, ..select_cols]

# SIR3-EcoGII
methyl_1 <- fread("/home/mbrothers/nanopore/201125_Turkey/modified_bases.aggregate07.6mA.bed",
                col.names = columns)
methyl_filtered_1 <- methyl_1[coverage > 10, ..select_cols]

# SIR2-EcoGII
methyl_2 <- fread("/home/mbrothers/nanopore/210917_Gorgonzola/modified_bases.aggregate05.6mA.bed",
                col.names = columns)
methyl_filtered_2 <- methyl_2[coverage > 10, ..select_cols]

# SIR4-EcoGII
methyl_3 <- fread("/home/mbrothers/nanopore/210614_Raja/modified_bases.aggregate.6mA.bed",
                col.names = columns)
methyl_filtered_3 <- methyl_3[coverage > 10, ..select_cols]

# PLOT CONTROL REGION
plot_loess(methyl_filtered_0, methyl_filtered_1, methyl_filtered_2, methyl_filtered_3, "III", 80e3, 105e3)
```

```
# with genes:
# abline(v = c(79162, 82275, 83620, 83997, 91324, 92418, 92777, 94270, 94621, 95763, 96281, 101191, 
#              101317, 101788, 102075, 103358, 103571, 104350))

# PLOT HML
plot_loess(methyl_filtered_0, methyl_filtered_1, methyl_filtered_2, methyl_filtered_3, "III", 0, 25e3)
```

```
# # with genes:
# abline(v = c(11146, 14849, 9706, 11082, 6479, 8326, 15798, 16880, 17290, 18561, 18816, 22106, 22429, 23379,
#              23584, 23925, 23523, 23981, 24032, 24325))
# # with telomere:
# abline(v = 1098)

# PLOT HMR
plot_loess(methyl_filtered_0, methyl_filtered_1, methyl_filtered_2, methyl_filtered_3, "III", 280e3, 305e3)
```

```
# # with genes:
# abline(v = c(292674, 292769, 294805, 294864, 288170, 289258, 297049, 298605, 286762, 287937, 280117, 286443,
#              301271, 302221, 304361, 305467))
# # with Ty elements:
# abline(v = c(291922, 292167, 291373, 291712, 295958, 296190, 295003, 295330), col = "red")
# # with tRNA:
# abline(v = c(295484, 295556), col = "green")
```

## SIR3-ECOGII single read methylation with nanopore sequencing

```
# PLOT FUNCTION
plot_binary <- function(data) {
  ggplot(data, aes(x = pos, y = read_id, color = methylation)) +
    geom_point(shape = 15) +
    theme(axis.text.y = element_blank(),
          axis.ticks.y = element_blank(),
          axis.title.y = element_blank(),
          axis.title.x = element_blank(),
          panel.grid = element_blank(),
          panel.background = element_blank(),
          text = element_text(size = 15, color = "black", family = "Arial"),
          legend.position = "top",
          legend.title = element_blank(),
          legend.key = element_blank()) +
    scale_color_manual(values = c("gray90", "mediumpurple4"), na.value = "white")
}

# DATA
mega_directory <- "/home/mbrothers/nanopore/210706_Fireworks/"
chr <- "III" #which chromosome?
barcode <- "09"

probs <- fread(sprintf("%schr%s_%s.txt", mega_directory, chr, barcode),
                    select = c(1, 2, 3, 4, 5),
                    col.names = c("read_id", "chrm", "strand", "pos", "mod_log_prob"))

#1. create a binary column; if 10^mod_log_prob is >0.8, set as methylated (m6A). if < 0.8, set as unmethylated (A)
#2. add start and end positions for each read_id
#3. find the average methylation of each read (for ordering on plot)
#4. order by strand, avg methyl and read_id
probs_filtered <- probs[, methylation := ifelse(10^mod_log_prob > 0.8, TRUE, FALSE)][
  , list(read_id, pos, methylation, strand)][
    , start_pos := min(pos), by = read_id][
      , end_pos := max(pos), by = read_id][
        , avg_methyl := mean(methylation == TRUE), by = read_id][
          order(-avg_methyl, read_id)]

# extract each unique read_id to set the order (in same order as in the data table to start with) for single read plots
read_names <- unique(probs_filtered$read_id)
probs_filtered$read_id = factor(probs_filtered$read_id, levels = read_names)

# PLOT CONTROL REGION
control_region <- c(95e3, 100e3)
control <- probs_filtered[start_pos <= control_region[1]][end_pos >= control_region[2]][
  pos %between% control_region]
plot_binary(control)
```

```
# PLOT HML
HML_region <- c(10.5e3, 15.5e3)
HML_E <- c(11237, 11268)
HML_I <- c(14600, 14711)

HML_all <- probs_filtered[start_pos <= HML_region[1]][end_pos >= HML_region[2]][
  pos %between% HML_region]
HML_all <- droplevels(HML_all)
hmlp <- plot_binary(HML_all)
hmlp
```

```
#plot with vertical lines for silencers and gene starts and stops
hmlp + geom_vline(xintercept = c(HML_E[1], HML_E[2], HML_I[1], HML_I[2], 13282, 13809, 12386, 13018))
```

```
# PLOT HMR
HMR_region <- c(291e3, 296e3)
HMR_E = c(292674, 292769)
HMR_I = c(294805, 294864)

HMR_all <- probs_filtered[start_pos <= HMR_region[1]][end_pos >= HMR_region[2]][
  pos %between% HMR_region]
HMR_all <- droplevels(HMR_all)
hmrp <- plot_binary(HMR_all)
hmrp
```

```
#plot with vertical lines for silencers and gene starts and stops
hmrp + geom_vline(xintercept = c(HMR_E[1], HMR_E[2], HMR_I[1], HMR_I[2], 293835, 294321, 293179, 293538))
```

# Supplemental Figures

## SIR3-M.ECOGII aggregate methylation with nanopore sequencing biological replicate

```
# LOESS PLOT FUNCTION
# d = data, ch = chromosome, b = beginning, e = end
plot_loess <- function(d0, d1, d2, ch, b, e){
  region_methyl_0 <- d0[chrom == ch & start > b & start < e]
  lo_methyl_0 <- loess(percentage ~ start, data = region_methyl_0, weights = region_methyl_0$coverage, enp.target = 100)
  
  region_methyl_1 <- d1[chrom == ch & start > b & start < e]
  lo_methyl_1 <- loess(percentage ~ start, data = region_methyl_1, weights = region_methyl_1$coverage, enp.target = 100)
  
  region_methyl_2 <- d2[chrom == ch & start > b & start < e]
  lo_methyl_2 <- loess(percentage ~ start, data = region_methyl_2, weights = region_methyl_2$coverage, enp.target = 100)
  
  plot(x=lo_methyl_0$x[order(lo_methyl_0$x)], y=lo_methyl_0$fitted[order(lo_methyl_0$x)],
       type = 'l', lwd=2, ylim = c(0,100), col = "black", frame.plot = FALSE,
       xlab = "", ylab = "")
  lines(x=lo_methyl_1$x[order(lo_methyl_1$x)], y=lo_methyl_1$fitted[order(lo_methyl_1$x)],
        type = 'l', lwd=2, ylim = c(0,100), col = "lightskyblue3")
  lines(x=lo_methyl_2$x[order(lo_methyl_2$x)], y=lo_methyl_2$fitted[order(lo_methyl_2$x)],
        type = 'l', lwd=2, ylim = c(0,100), col = "mediumpurple4")
  box(bty="l")
}

# DATA
columns <- c("chrom", "start", "end", "name", "score", 
             "strand", "startCodon", "stopCodon", "color", 
             "coverage", "percentage")
select_cols <- c("chrom", "start", "coverage", "percentage")

# sir3∆::EcoGII from main figure
methyl_0 <- fread("/home/mbrothers/nanopore/201125_Turkey/modified_bases.aggregate05.6mA.bed",
                col.names = columns)
methyl_filtered_0 <- methyl_0[coverage > 10, ..select_cols]

# SIR3-EcoGII from main figure
methyl_1 <- fread("/home/mbrothers/nanopore/201125_Turkey/modified_bases.aggregate07.6mA.bed",
                col.names = columns)
methyl_filtered_1 <- methyl_1[coverage > 10, ..select_cols]

# SIR3-EcoGII biological replicate
methyl_2 <- fread("/home/mbrothers/nanopore/201012_Doudna/modified_bases.aggregate00.6mA.bed",
                col.names = columns)
methyl_filtered_2 <- methyl_2[coverage > 10, ..select_cols]


# PLOT CONTROL REGION
plot_loess(methyl_filtered_0, methyl_filtered_1, methyl_filtered_2, "III", 80e3, 105e3)
```

```
# with genes:
# abline(v = c(79162, 82275, 83620, 83997, 91324, 92418, 92777, 94270, 94621, 95763, 96281, 101191, 
#              101317, 101788, 102075, 103358, 103571, 104350))

# PLOT HML
plot_loess(methyl_filtered_0, methyl_filtered_1, methyl_filtered_2, "III", 0, 25e3)
```

```
# # with genes:
# abline(v = c(11146, 14849, 9706, 11082, 6479, 8326, 15798, 16880, 17290, 18561, 18816, 22106, 22429, 23379,
#              23584, 23925, 23523, 23981, 24032, 24325))
# # with telomere:
# abline(v = 1098)

# PLOT HMR
plot_loess(methyl_filtered_0, methyl_filtered_1, methyl_filtered_2, "III", 280e3, 305e3)
```

```
# # with genes:
# abline(v = c(292674, 292769, 294805, 294864, 288170, 289258, 297049, 298605, 286762, 287937, 280117, 286443,
#              301271, 302221, 304361, 305467))
# # with Ty elements:
# abline(v = c(291922, 292167, 291373, 291712, 295958, 296190, 295003, 295330), col = "red")
# # with tRNA:
# abline(v = c(295484, 295556), col = "green")
```

## SIR3-M.ECOGII single read methylation with nanopore sequencing biological replicate

```
# PLOT FUNCTION
plot_binary <- function(data) {
  ggplot(data, aes(x = pos, y = read_id, color = methylation)) +
    geom_point(shape = 15) +
    theme(axis.text.y = element_blank(),
          axis.ticks.y = element_blank(),
          axis.title.y = element_blank(),
          axis.title.x = element_blank(),
          panel.grid = element_blank(),
          panel.background = element_blank(),
          text = element_text(size = 15, color = "black", family = "Arial"),
          legend.position = "top",
          legend.title = element_blank(),
          legend.key = element_blank()) +
    scale_color_manual(values = c("gray90", "mediumpurple4"), na.value = "white")
}

# DATA
mega_directory <- "/home/mbrothers/nanopore/201012_Doudna/"
chr <- "III" #which chromosome?
barcode <- "00"

probs <- fread(sprintf("%schr%s_%s.txt", mega_directory, chr, barcode),
                    select = c(1, 2, 3, 4, 5),
                    col.names = c("read_id", "chrm", "strand", "pos", "mod_log_prob"))

#1. create a binary column; if 10^mod_log_prob is >0.8, set as methylated (m6A). if < 0.8, set as unmethylated (A)
#2. add start and end positions for each read_id
#3. find the average methylation of each read (for ordering on plot)
#4. order by strand, avg methyl and read_id
probs_filtered <- probs[, methylation := ifelse(10^mod_log_prob > 0.8, TRUE, FALSE)][
  , list(read_id, pos, methylation, strand)][
    , start_pos := min(pos), by = read_id][
      , end_pos := max(pos), by = read_id][
        , avg_methyl := mean(methylation == TRUE), by = read_id][
          order(-avg_methyl, read_id)]

# extract each unique read_id to set the order (in same order as in the data table to start with) for single read plots
read_names <- unique(probs_filtered$read_id)
probs_filtered$read_id = factor(probs_filtered$read_id, levels = read_names)

# PLOT CONTROL REGION
control_region <- c(95e3, 100e3)
control <- probs_filtered[start_pos <= control_region[1]+1500][end_pos >= control_region[2]-1500][
  pos %between% control_region]
plot_binary(control)
```

```
# PLOT HML
HML_region <- c(10.5e3, 15.5e3)
HML_E <- c(11237, 11268)
HML_I <- c(14600, 14711)

HML_all <- probs_filtered[start_pos <= HML_region[1]+1500][end_pos >= HML_region[2]-1500][
  pos %between% HML_region]
HML_all <- droplevels(HML_all)
hmlp <- plot_binary(HML_all)
hmlp
```

```
#plot with vertical lines for silencers and gene starts and stops
hmlp + geom_vline(xintercept = c(HML_E[1], HML_E[2], HML_I[1], HML_I[2], 13282, 13809, 12386, 13018))
```

```
# PLOT HMR
HMR_region <- c(291e3, 296e3)
HMR_E = c(292674, 292769)
HMR_I = c(294805, 294864)

HMR_all <- probs_filtered[start_pos <= HMR_region[1]+1500][end_pos >= HMR_region[2]-1500][
  pos %between% HMR_region]
HMR_all <- droplevels(HMR_all)
hmrp <- plot_binary(HMR_all)
hmrp
```

```
#plot with vertical lines for silencers and gene starts and stops
hmrp + geom_vline(xintercept = c(HMR_E[1], HMR_E[2], HMR_I[1], HMR_I[2], 293835, 294321, 293179, 293538))
```

## SIR3-M.ECOGII linker preference

```
# PLOT FUNCTIONS
methyl_vs_nucs <- function (methyl_data, nuc_data) {
  plot(x=methyl_data$start, y=rep(-3,3,length.out=nrow(methyl_data)), 
       type="n", ylim=c(-3,4), 
       ylab="Scaled average methylation / occupancy",
       xlab="Chromosome position",
       frame.plot = FALSE)
  box(bty="l")
  #legend("topleft", c("Methylation", "Nucleosome occupancy"), 
         #col=c("mediumpurple4", "grey50"), lty=1, cex=1,
         #bty='n', lwd=3)
  
  # Methylation
  loMethyl <- loess(percentage/100 ~ start, 
                    data = methyl_data, 
                    weights = methyl_data$coverage, 
                    enp.target = 50)
  lines(x=loMethyl$x[order(loMethyl$x)], y=scale(loMethyl$fitted[order(loMethyl$x)]), 
        col="mediumpurple4", lwd=3)
  
  # Nucleosome occupancy
  loOccup <- loess(occupancy/max(occupancy) ~ start, 
                   data=nuc_data, 
                   enp.target = 50)
  lines(x=loOccup$x, y=scale(loOccup$fitted), col="grey60", lwd=3)
}

average_methylation_nucs <- function(nucs_data, meth_data){  
  LtoN <- which(diff(nucs_data$occupancy < 0.4) == -1)
  NtoL <- which(diff(nucs_data$occupancy < 0.4) == 1)
  nuc_rows <- data.table(start = c(1,LtoN), end = c(NtoL, nrow(nucs_data)))
  nuc_rows <- nuc_rows[(nuc_rows$end - nuc_rows$start) >=10,]
  
  avg_meth_nucl <- c()
  for (i in 1:nrow(nuc_rows)) {
    sub_nucs <- nucs_data[nuc_rows$start[i]:nuc_rows$end[i]]
    begin <- min(sub_nucs$start)
    finish <- max(sub_nucs$end)
    
    sub_meth <- meth_data[start > begin & start < finish]
    avg_meth_nucl[i] <- mean(sub_meth$percentage)
  }
  return(avg_meth_nucl)
}

average_methylation_linkers <- function(nucs_data, meth_data) {
  LtoN <- which(diff(nucs_data$occupancy < 0.4) == -1)
  NtoL <- which(diff(nucs_data$occupancy < 0.4) == 1)
  linker_rows <- data.table(start = NtoL, end = LtoN)
  linker_rows <- linker_rows[(linker_rows$end - linker_rows$start) >=10,]
  
  avg_meth_linker <- c()
  for (i in 1:nrow(linker_rows)) {
    sub_linkers <- nucs_data[linker_rows$start[i]:linker_rows$end[i]]
    begin <- min(sub_linkers$start)
    finish <- max(sub_linkers$end)
    
    sub_meth <- meth_data[start > begin & start < finish]
    avg_meth_linker[i] <- mean(sub_meth$percentage)
  }
  return(avg_meth_linker)
}

methyl_boxplot <- function (nucs_methyl, linkers_methyl) {  
  combined <- data.table(region = c(rep("nucleosomes",length(nucs_methyl)),
                                    rep("linkers",length(linkers_methyl))),
                         avg_methylation = c(nucs_methyl, linkers_methyl))
  
  ggplot(combined, aes(x = region, y = avg_methylation)) +
    geom_boxplot(fill = "lavender") +
    geom_point() +
    ylim(c(0,90)) +
    theme(
      panel.background = element_blank(),
      panel.grid.minor = element_blank(),
      panel.grid.major = element_blank(),
      axis.line = element_line(color = "black"),
      text = element_text(size = 20, color = "black", family = "Arial")) +
    labs(x = "region",
         y = "average % methylated reads")
  
}

# DATA
# methylation
meth <- fread("/home/mbrothers/nanopore/201012_Doudna/modified_bases.aggregate00.6mA.bed")
colnames(meth) <- c("chrom", "start", "end", "name", "score", 
                    "strand", "startCodon", "stopCodon", "color", 
                    "coverage", "percentage")
meth_cols <- c("chrom", "start", "coverage", "percentage")
filtered_meth <- meth[coverage > 10, ..meth_cols]

# nucleosome occupancy
nucs <- fread("/home/mbrothers/not_my_data/GSE97290_Henikoff_Chereji_2018/Chereji_Occupancy_rep1_singlebp.bedGraph")
colnames(nucs) <- c("chrom", "start", "end", "occupancy")

# CONTROL REGION
ctrl_meth <- filtered_meth[chrom == "III" & start > 96e3 & start < 99e3]
ctrl_nucs <- nucs[chrom == "chrIII" & start > 96e3 & start < 99e3]
ctrl_linkers_meth <- average_methylation_linkers(ctrl_nucs, ctrl_meth)
ctrl_nucs_meth <- average_methylation_nucs(ctrl_nucs, ctrl_meth)
methyl_vs_nucs(ctrl_meth, ctrl_nucs)
```

```
# HML
HML_nucs <- nucs[chrom == "chrIII" & start > 11e3 & start < 14e3]
HML_meth <- filtered_meth[chrom == "III" & start > 11e3 & start < 14e3]
HML_nucs_meth <- average_methylation_nucs(HML_nucs, HML_meth)
HML_linkers_meth <- average_methylation_linkers(HML_nucs, HML_meth)
methyl_vs_nucs(HML_meth, HML_nucs)
```

```
# abline(v = c(11146, 11082, 13282, 13809, 12386, 13018))

# HMR
HMR_nucs <- nucs[chrom == "chrIII" & start > 292e3 & start < 295e3]
HMR_meth <- filtered_meth[chrom == "III" & start > 292e3 & start < 295e3]
HMR_nucs_meth <- average_methylation_nucs(HMR_nucs, HMR_meth)
HMR_linkers_meth <- average_methylation_linkers(HMR_nucs, HMR_meth)
methyl_vs_nucs(HMR_meth, HMR_nucs)
```

```
# abline(v = c(292674, 292769, 294805, 294864, 293835, 294321, 293179, 293538))
```

```
#HML and HMR together: quasibinomial glm
all_linkers <- c(HMR_linkers_meth, HML_linkers_meth)
all_nucs <- c(HMR_nucs_meth, HML_nucs_meth)
methyl_boxplot(all_nucs, all_linkers)
```

```
all_together <- data.table(region = c(rep("nucleosome",length(all_nucs)),
                                      rep("linker",length(all_linkers))),
                                      avg_methylation = c(all_nucs, all_linkers))
all_together$avg_methylation <- all_together$avg_methylation / 100
m <- glm(avg_methylation ~ region,
         data = all_together,
         family = "quasibinomial")
summary(m)
```

```
## 
## Call:
## glm(formula = avg_methylation ~ region, family = "quasibinomial", 
##     data = all_together)
## 
## Deviance Residuals: 
##      Min        1Q    Median        3Q       Max  
## -0.36370  -0.07989   0.02405   0.12217   0.28004  
## 
## Coefficients:
##                  Estimate Std. Error t value Pr(>|t|)    
## (Intercept)       0.32818    0.08494   3.864 0.000513 ***
## regionnucleosome -0.56961    0.11640  -4.893 2.71e-05 ***
## ---
## Signif. codes:  0 '***' 0.001 '**' 0.01 '*' 0.05 '.' 0.1 ' ' 1
## 
## (Dispersion parameter for quasibinomial family taken to be 0.02809531)
## 
##     Null deviance: 1.58663  on 33  degrees of freedom
## Residual deviance: 0.90685  on 32  degrees of freedom
## AIC: NA
## 
## Number of Fisher Scoring iterations: 3
```

```
#plot(m)
```
