# Supplementary material for: Distinguishing between recruitment and spread of silent chromatin structures in Saccharomyces cerevisiae
Source: eLife. 2022 Jan 24;11:e75653. doi: 10.7554/eLife.75653 (PMC8830885; doi:10.7554/eLife.75653)
Supplement: Source code 5. [file elife-75653-code5.zip › Fig2.html]

Fig2


# Fig2

#### Molly Brothers

#### 10/06/21

## sir3-bahD-M.ECOGII aggregate methylation with nanopore sequencing

```
# LOESS PLOT FUNCTION
# d = data, ch = chromosome, b = beginning, e = end
plot_loess <- function(d0, d1, d2, d3, d4, d5, ch, b, e){
  region_methyl_0 <- d0[chrom == ch & start > b & start < e]
  lo_methyl_0 <- loess(percentage ~ start, data = region_methyl_0, weights = region_methyl_0$coverage, enp.target = 100)
  
  region_methyl_1 <- d1[chrom == ch & start > b & start < e]
  lo_methyl_1 <- loess(percentage ~ start, data = region_methyl_1, weights = region_methyl_1$coverage, enp.target = 100)
  
  region_methyl_2 <- d2[chrom == ch & start > b & start < e]
  lo_methyl_2 <- loess(percentage ~ start, data = region_methyl_2, weights = region_methyl_2$coverage, enp.target = 100)
  
  region_methyl_3 <- d3[chrom == ch & start > b & start < e]
  lo_methyl_3 <- loess(percentage ~ start, data = region_methyl_3, weights = region_methyl_3$coverage, enp.target = 100)
  
  region_methyl_4 <- d4[chrom == ch & start > b & start < e]
  lo_methyl_4 <- loess(percentage ~ start, data = region_methyl_4, weights = region_methyl_4$coverage, enp.target = 100)
  
  region_methyl_5 <- d5[chrom == ch & start > b & start < e]
  lo_methyl_5 <- loess(percentage ~ start, data = region_methyl_5, weights = region_methyl_5$coverage, enp.target = 100)
  
  plot(x=lo_methyl_0$x[order(lo_methyl_0$x)], y=lo_methyl_0$fitted[order(lo_methyl_0$x)], 
     type = 'l', lwd=2, ylim = c(0,100), col = "mediumpurple4", frame.plot = FALSE,
     xlab = "", ylab = "")
  lines(x=lo_methyl_1$x[order(lo_methyl_1$x)], y=lo_methyl_1$fitted[order(lo_methyl_1$x)],
      type = 'l', lwd=2, ylim = c(0,100), col = "lightskyblue3")
  lines(x=lo_methyl_2$x[order(lo_methyl_2$x)], y=lo_methyl_2$fitted[order(lo_methyl_2$x)],
      type = 'l', lwd=2, ylim = c(0,100), col = "black")
  lines(x=lo_methyl_3$x[order(lo_methyl_3$x)], y=lo_methyl_3$fitted[order(lo_methyl_3$x)],
      type = 'l', lwd=2, ylim = c(0,100), col = "gray40")
  lines(x=lo_methyl_4$x[order(lo_methyl_4$x)], y=lo_methyl_4$fitted[order(lo_methyl_4$x)],
      type = 'l', lwd=2, ylim = c(0,100), col = "deeppink")
  lines(x=lo_methyl_5$x[order(lo_methyl_5$x)], y=lo_methyl_5$fitted[order(lo_methyl_5$x)],
      type = 'l', lwd=2, ylim = c(0,100), col = "lightpink2")
  box(bty="l")
}

# DATA
columns <- c("chrom", "start", "end", "name", "score", 
             "strand", "startCodon", "stopCodon", "color", 
             "coverage", "percentage")
select_cols <- c("chrom", "start", "coverage", "percentage")

# SIR3-EcoGII
methyl_wt <- fread("/home/mbrothers/nanopore/210706_Fireworks/modified_bases.aggregate09.6mA.bed",
                col.names = columns)
methyl_filtered_wt <- methyl_wt[coverage > 10, ..select_cols]

methyl_wt2 <- fread("/home/mbrothers/nanopore/210730_Ashby/modified_bases.aggregate01.6mA.bed",
                col.names = columns)
methyl_filtered_wt2 <- methyl_wt2[coverage > 10, ..select_cols]

# sir3∆::EcoGII
methyl_D <- fread("/home/mbrothers/nanopore/210706_Fireworks/modified_bases.aggregate10.6mA.bed",
                col.names = columns)
methyl_filtered_D <- methyl_D[coverage > 10, ..select_cols]

methyl_D2 <- fread("/home/mbrothers/nanopore/210730_Ashby/modified_bases.aggregate02.6mA.bed",
                col.names = columns)
methyl_filtered_D2 <- methyl_D2[coverage > 10, ..select_cols]

# sir3-bah∆::EcoGII
methyl_bah <- fread("/home/mbrothers/nanopore/210706_Fireworks/modified_bases.aggregate11.6mA.bed",
                col.names = columns)
methyl_filtered_bah <- methyl_bah[coverage > 10, ..select_cols]

methyl_bah2 <- fread("/home/mbrothers/nanopore/210730_Ashby/modified_bases.aggregate04.6mA.bed",
                col.names = columns)
methyl_filtered_bah2 <- methyl_bah2[coverage > 10, ..select_cols]

# PLOT CONTROL REGION
plot_loess(methyl_filtered_wt, methyl_filtered_wt2, methyl_filtered_D, methyl_filtered_D2,
           methyl_filtered_bah, methyl_filtered_bah2, "III", 80e3, 105e3)
```

```
# with genes:
# abline(v = c(79162, 82275, 83620, 83997, 91324, 92418, 92777, 94270, 94621, 95763, 96281, 101191, 
#              101317, 101788, 102075, 103358, 103571, 104350))

# PLOT HML
plot_loess(methyl_filtered_wt, methyl_filtered_wt2, methyl_filtered_D, methyl_filtered_D2,
           methyl_filtered_bah, methyl_filtered_bah2, "III", 0, 25e3)
```

```
# with genes:
# abline(v = c(11146, 14849, 9706, 11082, 6479, 8326, 15798, 16880, 17290, 18561, 18816, 22106, 22429, 23379,
#              23584, 23925, 23523, 23981, 24032, 24325))
# # with telomere:
# abline(v = 1098)

# PLOT HMR
plot_loess(methyl_filtered_wt, methyl_filtered_wt2, methyl_filtered_D, methyl_filtered_D2,
           methyl_filtered_bah, methyl_filtered_bah2, "III", 280e3, 305e3)
```

```
# with genes:
# abline(v = c(292674, 292769, 294805, 294864, 288170, 289258, 297049, 298605, 286762, 287937, 280117, 286443,
#              301271, 302221, 304361, 305467))
# with Ty elements:
# abline(v = c(291922, 292167, 291373, 291712, 295958, 296190, 295003, 295330), col = "red")
# with tRNA:
# abline(v = c(295484, 295556), col = "green")

# PLOT TELOMERES
chromosomes <- c("I", "II", "III", "IV", "V", "VI", "VII", "VIII",
                 "IX", "X", "XI", "XII", "XIII", "XIV", "XV", "XVI")
right_ends <- c(230218, 813184, 316620, 1531933, 576874, 270161, 1090940, 562643,
                439888, 745751, 666816, 1078177, 924431, 765000, 1091291, 948010)

for (i in 1:16){
  plot_loess(methyl_filtered_wt, methyl_filtered_wt2, methyl_filtered_D, methyl_filtered_D2,
             methyl_filtered_bah, methyl_filtered_bah2, chromosomes[i], 0, 15e3)
}
```

```
for (i in 1:16){
  plot_loess(methyl_filtered_wt, methyl_filtered_wt2, methyl_filtered_D, methyl_filtered_D2,
             methyl_filtered_bah, methyl_filtered_bah2, chromosomes[i], right_ends[i]-15e3, right_ends[i])
}
```

## sir3-bahD-M.ECOGII single read methylation with nanopore sequencing

### HML and HMR single reads

```
# PLOT FUNCTION
plot_binary <- function(data) {
  ggplot(data, aes(x = pos, y = read_id, color = methylation)) +
    geom_point(shape = 15) +
    theme(axis.text.y = element_blank(),
          axis.ticks.y = element_blank(),
          axis.title.y = element_blank(),
          axis.title.x = element_blank(),
          panel.grid = element_blank(),
          panel.background = element_blank(),
          text = element_text(size = 15, color = "black", family = "Arial"),
          legend.position = "top",
          legend.title = element_blank(),
          legend.key = element_blank()) +
    scale_color_manual(values = c("gray90", "mediumpurple4"), na.value = "white")
}

# DATA
mega_directory <- "/home/mbrothers/nanopore/210706_Fireworks/"
chr <- "III" #which chromosome?
barcode <- "11"

probs <- fread(sprintf("%schr%s_%s.txt", mega_directory, chr, barcode),
                    select = c(1, 2, 3, 4, 5),
                    col.names = c("read_id", "chrm", "strand", "pos", "mod_log_prob"))

#1. create a binary column; if 10^mod_log_prob is >0.8, set as methylated (m6A). if < 0.8, set as unmethylated (A)
#2. add start and end positions for each read_id
#3. find the average methylation of each read (for ordering on plot)
#4. order by strand, avg methyl and read_id
probs_filtered <- probs[, methylation := ifelse(10^mod_log_prob > 0.8, TRUE, FALSE)][
  , list(read_id, pos, methylation, strand)][
    , start_pos := min(pos), by = read_id][
      , end_pos := max(pos), by = read_id][
        , avg_methyl := mean(methylation == TRUE), by = read_id][
          order(-avg_methyl, read_id)]

# extract each unique read_id to set the order (in same order as in the data table to start with) for single read plots
read_names <- unique(probs_filtered$read_id)
probs_filtered$read_id = factor(probs_filtered$read_id, levels = read_names)

# PLOT CONTROL REGION
control_region <- c(95e3, 100e3)
control <- probs_filtered[start_pos <= control_region[1]][end_pos >= control_region[2]][
  pos %between% control_region]
plot_binary(control)
```

```
# PLOT HML
HML_region <- c(10.5e3, 15.5e3)
HML_E <- c(11237, 11268)
HML_I <- c(14600, 14711)

HML_all <- probs_filtered[start_pos <= HML_region[1]][end_pos >= HML_region[2]][
  pos %between% HML_region]
HML_all <- droplevels(HML_all)
hmlp <- plot_binary(HML_all)
hmlp
```

```
# plot with vertical lines for silencers and gene starts and stops
hmlp + geom_vline(xintercept = c(HML_E[1], HML_E[2], HML_I[1], HML_I[2], 13282, 13809, 12386, 13018))
```

```
# plot with vertical line for HO cut site
# hmlp + geom_vline(xintercept = 13694)

# PLOT HMR
HMR_region <- c(291e3, 296e3)
HMR_E = c(292674, 292769)
HMR_I = c(294805, 294864)

HMR_all <- probs_filtered[start_pos <= HMR_region[1]][end_pos >= HMR_region[2]][
  pos %between% HMR_region]
HMR_all <- droplevels(HMR_all)
hmrp <- plot_binary(HMR_all)
hmrp
```

```
#plot with vertical lines for silencers and gene starts and stops
hmrp + geom_vline(xintercept = c(HMR_E[1], HMR_E[2], HMR_I[1], HMR_I[2], 293835, 294321, 293179, 293538))
```

### telomere 1L single reads

```
plot_binary <- function(data) {
  ggplot(data, aes(x = pos, y = read_id, color = methylation)) +
    geom_point(shape = 15) +
    theme(axis.text.y = element_blank(),
          axis.ticks.y = element_blank(),
          axis.title.y = element_blank(),
          axis.title.x = element_blank(),
          panel.grid = element_blank(),
          panel.background = element_blank(),
          text = element_text(size = 15, color = "black", family = "Arial"),
          legend.position = "top",
          legend.title = element_blank(),
          legend.key = element_blank()) +
    scale_color_manual(values = c("gray90", "mediumpurple4"), na.value = "white")
}

#### sir3∆::EcoGII ####
mega_directory <- "/home/mbrothers/nanopore/210706_Fireworks/"
chr <- "I" #which chromosome?
barcode <- "10"

probs <- fread(sprintf("%schr%s_%s.txt", mega_directory, chr, barcode),
                    select = c(1, 2, 3, 4, 5),
                    col.names = c("read_id", "chrm", "strand", "pos", "mod_log_prob"))

#1. create a binary column; if 10^mod_log_prob is >0.8, set as methylated (m6A). if < 0.8, set as unmethylated (A)
#2. add start and end positions for each read_id
#3. find the average methylation of each read (for ordering on plot)
#4. order by strand, avg methyl and read_id
probs_filtered <- probs[, methylation := ifelse(10^mod_log_prob > 0.8, TRUE, FALSE)][
  , list(read_id, pos, methylation, strand)][
    , start_pos := min(pos), by = read_id][
      , end_pos := max(pos), by = read_id][
        , avg_methyl := mean(methylation == TRUE), by = read_id][
          order(-avg_methyl, read_id)]

# extract each unique read_id to set the order (in same order as in the data table to start with) for single read plots
read_names <- unique(probs_filtered$read_id)
probs_filtered$read_id = factor(probs_filtered$read_id, levels = read_names)

# plot
tel1L_region <- c(0, 10e3)
tel1L <- probs_filtered[start_pos <= tel1L_region[1]+500][end_pos >= tel1L_region[2]][
  pos %between% tel1L_region]
plot_binary(tel1L)
```

```
#### SIR3-EcoGII ####
mega_directory <- "/home/mbrothers/nanopore/210706_Fireworks/"
chr <- "I" #which chromosome?
barcode <- "09"

probs <- fread(sprintf("%schr%s_%s.txt", mega_directory, chr, barcode),
                    select = c(1, 2, 3, 4, 5),
                    col.names = c("read_id", "chrm", "strand", "pos", "mod_log_prob"))

#1. create a binary column; if 10^mod_log_prob is >0.8, set as methylated (m6A). if < 0.8, set as unmethylated (A)
#2. add start and end positions for each read_id
#3. find the average methylation of each read (for ordering on plot)
#4. order by strand, avg methyl and read_id
probs_filtered <- probs[, methylation := ifelse(10^mod_log_prob > 0.8, TRUE, FALSE)][
  , list(read_id, pos, methylation, strand)][
    , start_pos := min(pos), by = read_id][
      , end_pos := max(pos), by = read_id][
        , avg_methyl := mean(methylation == TRUE), by = read_id][
          order(-avg_methyl, read_id)]

# extract each unique read_id to set the order (in same order as in the data table to start with) for single read plots
read_names <- unique(probs_filtered$read_id)
probs_filtered$read_id = factor(probs_filtered$read_id, levels = read_names)

# plot
tel1L_region <- c(0, 10e3)
tel1L <- probs_filtered[start_pos <= tel1L_region[1]+500][end_pos >= tel1L_region[2]][
  pos %between% tel1L_region]
plot_binary(tel1L)
```

```
#### sir3-bah∆-EcoGII ####
mega_directory <- "/home/mbrothers/nanopore/210706_Fireworks/"
chr <- "I" #which chromosome?
barcode <- "11"

probs <- fread(sprintf("%schr%s_%s.txt", mega_directory, chr, barcode),
                    select = c(1, 2, 3, 4, 5),
                    col.names = c("read_id", "chrm", "strand", "pos", "mod_log_prob"))

#1. create a binary column; if 10^mod_log_prob is >0.8, set as methylated (m6A). if < 0.8, set as unmethylated (A)
#2. add start and end positions for each read_id
#3. find the average methylation of each read (for ordering on plot)
#4. order by strand, avg methyl and read_id
probs_filtered <- probs[, methylation := ifelse(10^mod_log_prob > 0.8, TRUE, FALSE)][
  , list(read_id, pos, methylation, strand)][
    , start_pos := min(pos), by = read_id][
      , end_pos := max(pos), by = read_id][
        , avg_methyl := mean(methylation == TRUE), by = read_id][
          order(-avg_methyl, read_id)]

# extract each unique read_id to set the order (in same order as in the data table to start with) for single read plots
read_names <- unique(probs_filtered$read_id)
probs_filtered$read_id = factor(probs_filtered$read_id, levels = read_names)

# plot
tel1L_region <- c(0, 10e3)
tel1L <- probs_filtered[start_pos <= tel1L_region[1]+500][end_pos >= tel1L_region[2]][
  pos %between% tel1L_region]
plot_binary(tel1L)
```

### telomere 2L single reads

```
plot_binary <- function(data) {
  ggplot(data, aes(x = pos, y = read_id, color = methylation)) +
    geom_point(shape = 15) +
    theme(axis.text.y = element_blank(),
          axis.ticks.y = element_blank(),
          axis.title.y = element_blank(),
          axis.title.x = element_blank(),
          panel.grid = element_blank(),
          panel.background = element_blank(),
          text = element_text(size = 15, color = "black", family = "Arial"),
          legend.position = "top",
          legend.title = element_blank(),
          legend.key = element_blank()) +
    scale_color_manual(values = c("gray90", "mediumpurple4"), na.value = "white")
}

#### sir3∆::EcoGII ####
mega_directory <- "/home/mbrothers/nanopore/210706_Fireworks/"
chr <- "II" #which chromosome?
barcode <- "10"

probs <- fread(sprintf("%schr%s_%s.txt", mega_directory, chr, barcode),
                    select = c(1, 2, 3, 4, 5),
                    col.names = c("read_id", "chrm", "strand", "pos", "mod_log_prob"))

#1. create a binary column; if 10^mod_log_prob is >0.8, set as methylated (m6A). if < 0.8, set as unmethylated (A)
#2. add start and end positions for each read_id
#3. find the average methylation of each read (for ordering on plot)
#4. order by strand, avg methyl and read_id
probs_filtered <- probs[, methylation := ifelse(10^mod_log_prob > 0.8, TRUE, FALSE)][
  , list(read_id, pos, methylation, strand)][
    , start_pos := min(pos), by = read_id][
      , end_pos := max(pos), by = read_id][
        , avg_methyl := mean(methylation == TRUE), by = read_id][
          order(-avg_methyl, read_id)]

# extract each unique read_id to set the order (in same order as in the data table to start with) for single read plots
read_names <- unique(probs_filtered$read_id)
probs_filtered$read_id = factor(probs_filtered$read_id, levels = read_names)

# plot
tel2L_region <- c(0, 10e3)
tel2L <- probs_filtered[start_pos <= tel2L_region[1]+500][end_pos >= tel2L_region[2]][
  pos %between% tel2L_region]
plot_binary(tel2L)
```

```
#### SIR3-EcoGII ####
mega_directory <- "/home/mbrothers/nanopore/210706_Fireworks/"
chr <- "II" #which chromosome?
barcode <- "09"

probs <- fread(sprintf("%schr%s_%s.txt", mega_directory, chr, barcode),
                    select = c(1, 2, 3, 4, 5),
                    col.names = c("read_id", "chrm", "strand", "pos", "mod_log_prob"))

#1. create a binary column; if 10^mod_log_prob is >0.8, set as methylated (m6A). if < 0.8, set as unmethylated (A)
#2. add start and end positions for each read_id
#3. find the average methylation of each read (for ordering on plot)
#4. order by strand, avg methyl and read_id
probs_filtered <- probs[, methylation := ifelse(10^mod_log_prob > 0.8, TRUE, FALSE)][
  , list(read_id, pos, methylation, strand)][
    , start_pos := min(pos), by = read_id][
      , end_pos := max(pos), by = read_id][
        , avg_methyl := mean(methylation == TRUE), by = read_id][
          order(-avg_methyl, read_id)]

# extract each unique read_id to set the order (in same order as in the data table to start with) for single read plots
read_names <- unique(probs_filtered$read_id)
probs_filtered$read_id = factor(probs_filtered$read_id, levels = read_names)

# plot
tel2L_region <- c(0, 10e3)
tel2L <- probs_filtered[start_pos <= tel2L_region[1]+500][end_pos >= tel2L_region[2]][
  pos %between% tel2L_region]
plot_binary(tel2L)
```

```
#### sir3-bah∆-EcoGII ####
mega_directory <- "/home/mbrothers/nanopore/210706_Fireworks/"
chr <- "II" #which chromosome?
barcode <- "11"

probs <- fread(sprintf("%schr%s_%s.txt", mega_directory, chr, barcode),
                    select = c(1, 2, 3, 4, 5),
                    col.names = c("read_id", "chrm", "strand", "pos", "mod_log_prob"))

#1. create a binary column; if 10^mod_log_prob is >0.8, set as methylated (m6A). if < 0.8, set as unmethylated (A)
#2. add start and end positions for each read_id
#3. find the average methylation of each read (for ordering on plot)
#4. order by strand, avg methyl and read_id
probs_filtered <- probs[, methylation := ifelse(10^mod_log_prob > 0.8, TRUE, FALSE)][
  , list(read_id, pos, methylation, strand)][
    , start_pos := min(pos), by = read_id][
      , end_pos := max(pos), by = read_id][
        , avg_methyl := mean(methylation == TRUE), by = read_id][
          order(-avg_methyl, read_id)]

# extract each unique read_id to set the order (in same order as in the data table to start with) for single read plots
read_names <- unique(probs_filtered$read_id)
probs_filtered$read_id = factor(probs_filtered$read_id, levels = read_names)

# plot
tel2L_region <- c(0, 10e3)
tel2L <- probs_filtered[start_pos <= tel2L_region[1]+500][end_pos >= tel2L_region[2]][
  pos %between% tel2L_region]
plot_binary(tel2L)
```
