# Supplementary material for: Distinguishing between recruitment and spread of silent chromatin structures in Saccharomyces cerevisiae
Source: eLife. 2022 Jan 24;11:e75653. doi: 10.7554/eLife.75653 (PMC8830885; doi:10.7554/eLife.75653)
Supplement: Source code 5. [file elife-75653-code5.zip › Fig3.html]

Fig3


# Fig3

#### Molly Brothers

#### 10/25/2021

## SIR3-M.ECOGII overexpression aggregate methylation with nanopore sequencing

```
# LOESS PLOT FUNCTION
# d = data, ch = chromosome, b = beginning, e = end
plot_loess <- function(d0, d1, d2, d3, ch, b, e){
  region_methyl_0 <- d0[chrom == ch & start > b & start < e]
  lo_methyl_0 <- loess(percentage ~ start, data = region_methyl_0, weights = region_methyl_0$coverage, enp.target = 100)
  
  region_methyl_1 <- d1[chrom == ch & start > b & start < e]
  lo_methyl_1 <- loess(percentage ~ start, data = region_methyl_1, weights = region_methyl_1$coverage, enp.target = 100)

  region_methyl_2 <- d2[chrom == ch & start > b & start < e]
  lo_methyl_2 <- loess(percentage ~ start, data = region_methyl_2, weights = region_methyl_2$coverage, enp.target = 100) 
  
  region_methyl_3 <- d3[chrom == ch & start > b & start < e]
  lo_methyl_3 <- loess(percentage ~ start, data = region_methyl_3, weights = region_methyl_3$coverage, enp.target = 100)
  
  plot(x=lo_methyl_0$x[order(lo_methyl_0$x)], y=lo_methyl_0$fitted[order(lo_methyl_0$x)], 
     type = 'l', lwd=2, ylim = c(0,100), col = "gray40", frame.plot = FALSE,
     xlab = "", ylab = "")
  lines(x=lo_methyl_1$x[order(lo_methyl_1$x)], y=lo_methyl_1$fitted[order(lo_methyl_1$x)],
      type = 'l', lwd=2, ylim = c(0,100), col = "black")  
  lines(x=lo_methyl_2$x[order(lo_methyl_2$x)], y=lo_methyl_2$fitted[order(lo_methyl_2$x)],
      type = 'l', lwd=2, ylim = c(0,100), col = "firebrick2")
  lines(x=lo_methyl_3$x[order(lo_methyl_3$x)], y=lo_methyl_3$fitted[order(lo_methyl_3$x)],
      type = 'l', lwd=2, ylim = c(0,100), col = "lightcoral")  
  box(bty="l")
}

# DATA
columns <- c("chrom", "start", "end", "name", "score", 
             "strand", "startCodon", "stopCodon", "color", 
             "coverage", "percentage")
select_cols <- c("chrom", "start", "coverage", "percentage")

# SIR3-ecoGII empty vector
methyl_ev1 <- fread("/home/mbrothers/nanopore/211022_Bonus/modified_bases.aggregate03.6mA.bed",
                col.names = columns)
methyl_filtered_ev1 <- methyl_ev1[coverage > 10, ..select_cols]

methyl_ev2 <- fread("/home/mbrothers/nanopore/211101_TakeFive/modified_bases.aggregate05.6mA.bed",
                col.names = columns)
methyl_filtered_ev2 <- methyl_ev2[coverage > 10, ..select_cols]

# SIR3-ecoGII overexpression
methyl_oe1 <- fread("/home/mbrothers/nanopore/211022_Bonus/modified_bases.aggregate04.6mA.bed",
                col.names = columns)
methyl_filtered_oe1 <- methyl_oe1[coverage > 10, ..select_cols]

methyl_oe2 <- fread("/home/mbrothers/nanopore/211101_TakeFive/modified_bases.aggregate06.6mA.bed",
                col.names = columns)
methyl_filtered_oe2 <- methyl_oe2[coverage > 10, ..select_cols]

# PLOT CONTROL REGION
plot_loess(methyl_filtered_ev1, methyl_filtered_ev2, methyl_filtered_oe1, methyl_filtered_oe2, "III", 80e3, 105e3)
```

```
# with genes:
# abline(v = c(79162, 82275, 83620, 83997, 91324, 92418, 92777, 94270, 94621, 95763, 96281, 101191, 
#              101317, 101788, 102075, 103358, 103571, 104350))

# PLOT HML
plot_loess(methyl_filtered_ev1, methyl_filtered_ev2, methyl_filtered_oe1, methyl_filtered_oe2, "III", 0, 25e3)
```

```
# with genes:
# abline(v = c(11146, 14849, 9706, 11082, 6479, 8326, 15798, 16880, 17290, 18561, 18816, 22106, 22429, 23379,
#              23584, 23925, 23523, 23981, 24032, 24325))
# # with telomere:
# abline(v = 1098)

# PLOT HMR
plot_loess(methyl_filtered_ev1, methyl_filtered_ev2, methyl_filtered_oe1, methyl_filtered_oe2, "III", 280e3, 305e3)
```

```
# with genes:
# abline(v = c(292674, 292769, 294805, 294864, 288170, 289258, 297049, 298605, 286762, 287937, 280117, 286443,
#              301271, 302221, 304361, 305467))
# with Ty elements:
# abline(v = c(291922, 292167, 291373, 291712, 295958, 296190, 295003, 295330), col = "red")
# with tRNA:
# abline(v = c(295484, 295556), col = "green")

# PLOT TELOMERES
chromosomes <- c("I", "II", "III", "IV", "V", "VI", "VII", "VIII",
                 "IX", "X", "XI", "XII", "XIII", "XIV", "XV", "XVI")
right_ends <- c(230218, 813184, 316620, 1531933, 576874, 270161, 1090940, 562643,
                439888, 745751, 666816, 1078177, 917000, 765000, 1091291, 948010)
for(i in 1:16){
  plot_loess(methyl_filtered_ev1, methyl_filtered_ev2, methyl_filtered_oe1, methyl_filtered_oe2, chromosomes[i], 0, 15e3)
}
```

```
for(i in 1:16){
  plot_loess(methyl_filtered_ev1, methyl_filtered_ev2, methyl_filtered_oe1, methyl_filtered_oe2, chromosomes[i], right_ends[i]-15e3, right_ends[i])
}
```
